# A novel radiosensitizer α-sulfoquinovosyl-acylpropanediol (SQAP) inhibits DNA repair pathways and sensitize cells to cancer chemotherapeutic agents

**DOI:** 10.1038/s41598-025-25524-0

**Published:** 2025-11-24

**Authors:** Junko Maeda, Shigeaki Sunada, Takaomi Fukuhara, Takamitsu A. Kato

**Affiliations:** 1 Department of Environmental and Radiological Health Sciences, Colorado State University, Fort Collins, CO USA; 2 Juntendo Advanced Research Institute for Health Sciences, Juntendo University, Tokyo, Japan; 3 M. T. 3 (Malignant Tumor Treatment Technologies) Inc, Osaka, Japan

**Keywords:** Α-sulfoquinovosyl-acylpropanediol, DNA repair, Chemotherapeutic agents, Cancer, Chemosensitizer, Chemotherapy, Radiotherapy, DNA damage and repair

## Abstract

Sulfoglycolipid, α-sulfoquinovosyl-acylpropanediol (SQAP) is a novel veterinary radiosensitizer, which is known to cause angiogenesis alteration and sensitizes hypoxic tumors in the in vivo animal model. We examined the SQAP radio/chemo-sensitization mechanisms from DNA repair with canine cancer cell lines and Chinese hamster cell lines. Previous studies have shown that SQAP radiosensitization was limited to in vivo xenograft models, but we found SQAP sensitized cells to radiation in vitro cell culture system. SQAP sensitized canine osteosarcoma and melanoma cell lines to gamma-ray irradiation in normoxia or hypoxia conditions. This result suggested that SQAP was expected to affect the repair of DNA damage induced by ionizing radiation and enhanced cellular radiosensitivity. To further identify potential mechanisms of radiosensitization, we utilized several assays to determine DNA repair inhibition by SQAP. SQAP treatment inhibited NHEJ and HR activity measured by EJ5-GFP and DR-GFP assays. SQAP treatment reduced the spontaneous sister chromatid exchange formation in CHO wild type and EM9 (XRCC1 mutant). On the other hand, 51D1 (rad51d mutant, homologous recombination (HR) repair deficient) showed no reduction. In vitro topoisomerase assay revealed SQAP disrupted topoisomerase I and II alpha activities. SQAP sensitized series of chemotherapeutic agents including doxorubicin, carboplatin, bleomycin, camptothecin, etoposide, methyl methanesulfonate, cisplatin, mitomycin C, and Taxol in canine tumor cells and V79 cells. These results suggest that broad inhibition of DNA repair may play a role in SQAP induced radiosensitization and chemosensitization.

## Introduction

Alpha-sulfoquinovosylacyl-1,3-propanediol (SQAP) is a synthetic derivative of the sulfoglycolipid natural product isolated from sea urchins and marine algae^1,2^. SQAP consists of a glucose molecule with a sulfone group and a stearic acid molecule connected by a propanediol. It was synthesized in preparation of clinical studies from α-SQMG, the prototype of SQAP, which was found to exhibit the radiosensitization effect in vivo mouse models^3,4^. The radiosensitization effect of SQAP has been reported in mouse xenograft models of human lung cancer, prostate cancer and malignant mesothelioma^5–7^. SQAP has been tested in clinical settings in dogs and cats with cancer receiving radiation therapy, and importantly adverse systemic events were not detected^8,9^. SQAP was approved by the Ministry of Agriculture, Forestry and Fisheries (MAFF) in Japan as a radiosensitizer for veterinary clinics in 2023 and is currently being applied to treat cancer in pet animals.

Hypoxic tumors are more resistant to radiotherapy because radiation-induced DNA damage is dependent on oxygen^10^. The mechanism underlying radiosensitization by SQAP has been attributed to several mechanisms that improve the hypoxic tumor microenvironment. An in vivo mouse study has shown that SQAP promotes oxygen dissociation from hemoglobin and tumor perfusion immediately after administration, temporarily reoxygenating the solid cancer tissues and enhancing radiation efficacy^5^. Another reason for SQAP radiosensitization likely involves its antiangiogenic property^3^. SQAP has been shown to induce tumor vascular normalization after radiation therapy in human colon adenocarcinoma and prostate cancer xenograft models, promoting tumor oxygenation and enhancing the efficiency of subsequent radiation treatments^4,7^. Furthermore, SQAP reduced the expression of hypoxia inducible factor-1α (HIF-1α) in tumors treated with combined radiation and SQAP in a human malignant mesothelioma xenograft model, suggesting that the interaction between radiation therapy and SQAP induced tumor reoxygenation^6^.

Other possible targets that may contribute as hypoxic sensitizers include histone deacetylase 1 (HDAC1)^11^. While SQAP does not inhibit HDAC1 enzymatically, it reduces HDAC1 expression under hypoxic conditions. Our previous study presented that a HDAC inhibitor selectively sensitizes cancer cells to radiation, but not normal cells, by disrupting of DNA repair^12^. Since radiation exposure causes DNA damage, typical radiosensitizers have the ability to disrupt DNA repair machinery and enhances radiation-induced cell death. We have identified that SQAP has poly (ADP-ribose) polymerase (PARP) and poly (ADP-ribose) glycohydrolase (PARG) inhibitory effect, which may lead to synthetic lethality in homologous recombination deficient cells, including BRCA2 mutant cancers^13^. Other investigations revealed that multiple targets, including focal adhesion kinase (FAK) and DNA polymerases, were inhibited by SQAP treatment^1,14^.

Despite these findings and clinical trials in veterinary clinics, the relative effect of SQAP of DNA repair underlying the radiosensitization mechanism remains largely unexplored. Ionizing radiation (IR) and most chemotherapeutic drugs can cause various DNA damages, and cells have multiple DNA repair pathways to repair these damages. DNA double strand breaks (DSBs) are critical lesions because failure to repair DSBs could be directly linked to the formation of lethal chromosomal aberrations^15^. Various other DNA lesions can disrupt replication and, if poorly or incompletely repaired, also lead DSBs. The two main pathways are involved in the repair of DSBs: non homologous end joining (NHEJ) and homologous recombination (HR)^16^. The components involved in the DNA repair machinery greatly influence cellular radiosensitivity and chemosensitivity^17,18^. Therefore, in this study, we investigated DNA repair-mediated SQAP radiosensitization as a potentially important mechanism. We first investigated whether SQAP treatment enhances cellular radiosensitivity and chemosensitivity through the inhibition of DNA repair using canine cancer cell lines and Chinese hamster cell lines. To elucidate the mechanisms behind SQAP-induced radiosensitization, we conducted a series of assays, focusing on DNA repair processes, such as NHEJ and HR.

## Materials and methods

### Cell lines and culture

Chinese hamster lung origin V79, and ovary origin CHO wild type and DNA repair deficient mutant EM9^19^, xrs5^20^ and 51D1^21^ were kindly supplied by Dr. Joel Bedford of Colorado State University (Fort Collins, CO) and Dr. Larry Thompson of Lawrence Livermore National Laboratory (Livermore, CA). Canine osteosarcoma cell lines (Abrams, D17 and Moresco), canine melanoma cell lines (CML-6M, CML-10C2, and Jones), and canine soft tissue sarcoma (STSA-1) were kindly supplied by Dr. Douglas Thamm of Colorado State University^22^. HeLa cell line was obtained from RIKEN BRC (Tsukuba, Japan). Cells were cultured in alpha-MEM supplemented with 10% heat inactivated Fetal Bovine Serum (Sigma-Aldrich, St. Louis, MO, USA), 1% antibiotics (Anti-Anti; Invitrogen, Grand Island, NY, USA) and maintained in 37 °C incubators with 5% CO_2_ and humidity.

### Chemicals

SQAP was provided by M.T.3, Inc. SQAP was diluted in 0.9% NaCl and used as 10–30 µM final concentration in media. Doxorubicin hydrochloride (DOX), carboplatin (CARBO) and lomustine (CCNU) were purchased from Enzo (Farmingdale, NY, USA). Chlorambucil (CBL) was purchased from Tokyo chemical industry. Mitoxantrone dihydrochloride (MTX) and prednisolone were purchased from Selleck Chemicals (Houston, TX, USA). Camptothecin, etoposide, cisplatin, methyl methanesulfonate (MMS), and bleomycin were obtained from Sigma-Aldrich. Mitomycin C (MMC) was obtained from Funakoshi (Tokyo, Japan). Taxol was obtained from Nippon Kayaku (Tokyo, Japan).

### Irradiation in normoxia and hypoxia

Irradiation was conducted by JL Shepard Mark I ^137^Cs irradiator (nominal 6000 Ci) with dose rate of approximately 2.5 Gy per minute at room temperature. For hypoxia irradiation, cell cultures were placed in Aneropak Kenki system (Mitsubishi Gas Chemical, Tokyo, Japan) with oxygen meter to confirm pO_2_ below 0.1%. Cells were kept in hypoxia for 3 h before irradiation^23^.

### Colony formation assay

Sensitivity to radiation or chemical reagents was assessed through colony formation assays. After trypsinized single cells were plated to form 20–200 colonies per dish and then treated with SQAP for 3 h in normoxia or hypoxia, the cells were irradiated. For drug sensitivity study, trypsinized cells were plated and then treated with various dosage of testing agents. After seven days of incubation, colonies were fixed, stained and scored^24^. Regression curves were drawn from survival fraction, and D_10_ values (doses to achieve 10% cell survival) were calculated by Prism 8 (GraphPad software, San Diego, CA, USA) from at least three independent experiments. Oxygen enhancement ratio (OER) and sensitization enhancement ratio (SER) values were calculated from D_10_ values. OER values were calculated from the D_10_ value in hypoxia divided by the D_10_ value in normoxic condition. SER values were calculated from the D_10_ value without SQAP divided by the D_10_ value with SQAP.

### DNA repair reporter assay

HR and NHEJ activities were assessed using the DR-GFP reporter system^25^ and the EJ5-GFP reporter system^26^, respectively. pDRGFP and pCBASceI were gifts from Dr. Maria Jasin (Addgene plasmid #26475 and #26477). pimEJ5GFP was a gift from Dr. Jeremy Stark (Addgene plasmid # 44026). The assays were performed as previously described^27^. Briefly, HeLa cells expressing DR-GFP, or EJ5-GFP were transfected with a plasmid encoding an I-SceI endonuclease using FuGENE HD transfection reagent (Promega, Madison, WI, USA). HR and NHEJ frequencies were analyzed by counting GFP positive cells with a FACS Lyric flow cytometer (BD Biosciences, San Jose, CA, USA) at 48 h post-transfection. Drug treatment was performed 3 h after transfection, and medium was replaced with the drug until FACS analysis. Relative HR and NHEJ efficiencies were calculated by normalizing to control cell values. A total of 50,000 cells were counted for each condition.

### Sister chromatid exchange assay

CHO cells were synchronized into G1 phase using mitotic shake-off method^28^. Cells in the G1 phase were irradiated, and 10 µM of BrdU was added. After 20 h, colcemid was added for 6 h to arrest cells in metaphase. Collected cells were treated with hypotonic KCl solution and fixed using methanol: acetic acid (3:1) as the standard method^29^. Metaphase chromosome spread was prepared on glass slides and treated with 5 µg/mL of Hoechst 33258 solution for 15 min and exposed to UVB for 15 min and treated with 2X SSC solution at 80 °C for 15 min. Cells were stained with 5% Giemsa stain solution. At least 50 metaphase spreads were analyzed for SCE scoring per experiment.

### Chromosome aberration assay

Two hours after mitotic shake-off and replating, CHO cells in the G1 phase were irradiated. After 10 h, colcemid was added for 6 h to collect metaphase cells. Chromosome spreads were prepared as sister chromatid exchange assay and stained with a 5% Giemsa stain. At least 50 metaphase spreads were scored in three independent experiments.

### G2 premature chromosome condensation (PCC) assay

Following treatment with SQAP for 1 h, exponentially growing CHO cells were irradiated and treated with 50 nM of Calyculin A (Sigma-Aldrich) for 30 min as previously described^30^. Cells were then fixed and stained as chromosome aberration analysis. Under a Zeiss Axioskop microscope, chromatid gaps, breaks, iso-breaks and exchanges were all scored as G2-PCC chromatid breaks. At least 50 PCC-spreads were scored in three independent experiments.

### Topoisomerase assay

For topoisomerase (Topo) I assay, a mixture of 125 ng of supercoiled pHOT1 DNA, 1U of Topo I enzyme, and various concentrations of SQAP in 1 mM Tris-HCl (pH7.9), 0.1 mM EDTA, 0.015 M NaCl, 0.01% BSA, 0.01 mM Spermidine and 0.5% glycerol was incubated at 37 °C for 30 min. For Topo II alpha assay, a mixture of 10 µL containing of 50 ng of kinetochore DNA in 0.05 M Tris-HCl (pH8), 1U of Topo II alpha, various concentrations of SQAP in 0.15 M NaCl, 10 mM MgCl_2_, 0.5mM DTT, 30 µg/mL of BSA, 2 mM ATP was incubated at 37 °C for 30 min. After the reaction was terminated, electrophoresis was conducted at 50 V for 3 h, followed by ethidium bromide staining. DNA images were obtained by a Bio-Rad imager (Bio-Rad, Hercules, CA, USA) and quantified with Image Lab software (Bio-Rad).

### Statistical analysis

Experiments were conducted as three or more biologically independent experiments. Data points were expressed as the mean with standard errors of the means. Statistical significance was determined by unpaired t-test or one-way analysis of variance (ANOVA) and Turkey’s multiple comparison test using GraphPad Prism 8. Differences with *P* values of less than 0.05 were considered statistically significant.

## Results

### SQAP sensitizes canine cancer cells to ionizing radiation in vitro

**Fig. 1:**
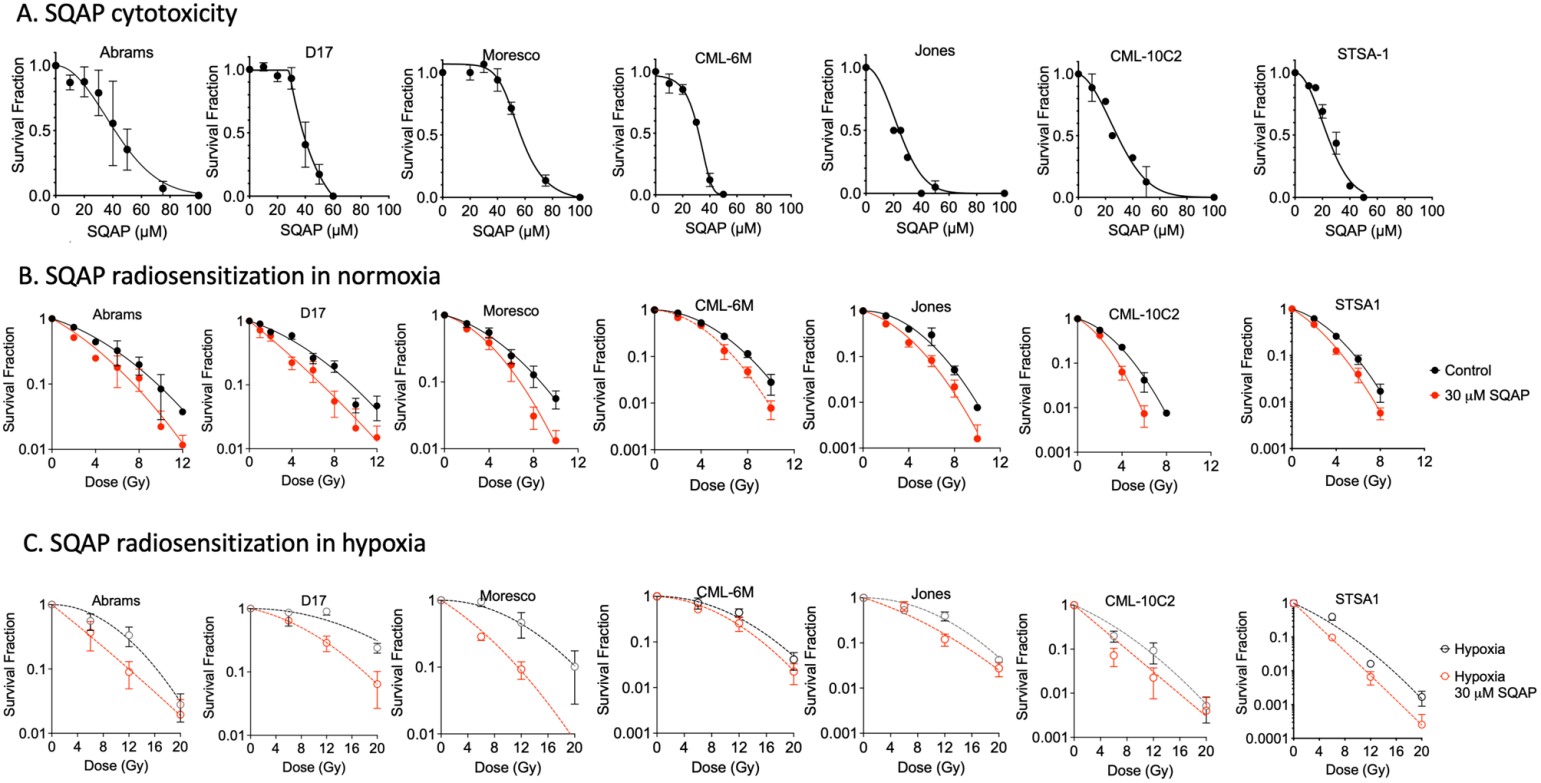
SQAP sensitizes canine cancer cells to ionizing radiation. (**A**) SQAP cytotoxicity obtained by clonogenic survival of cells treated with SQAP. (**B**) Clonogenic survival of cells treated with 30 µM SQAP in normoxia and (**C**) in hypoxia. All data are mean of at least three independent experiments except Jones and CML-6M data of cytotoxicity represent two independent experiments. Error bars indicate standard error of the mean.

**Fig. 2:**
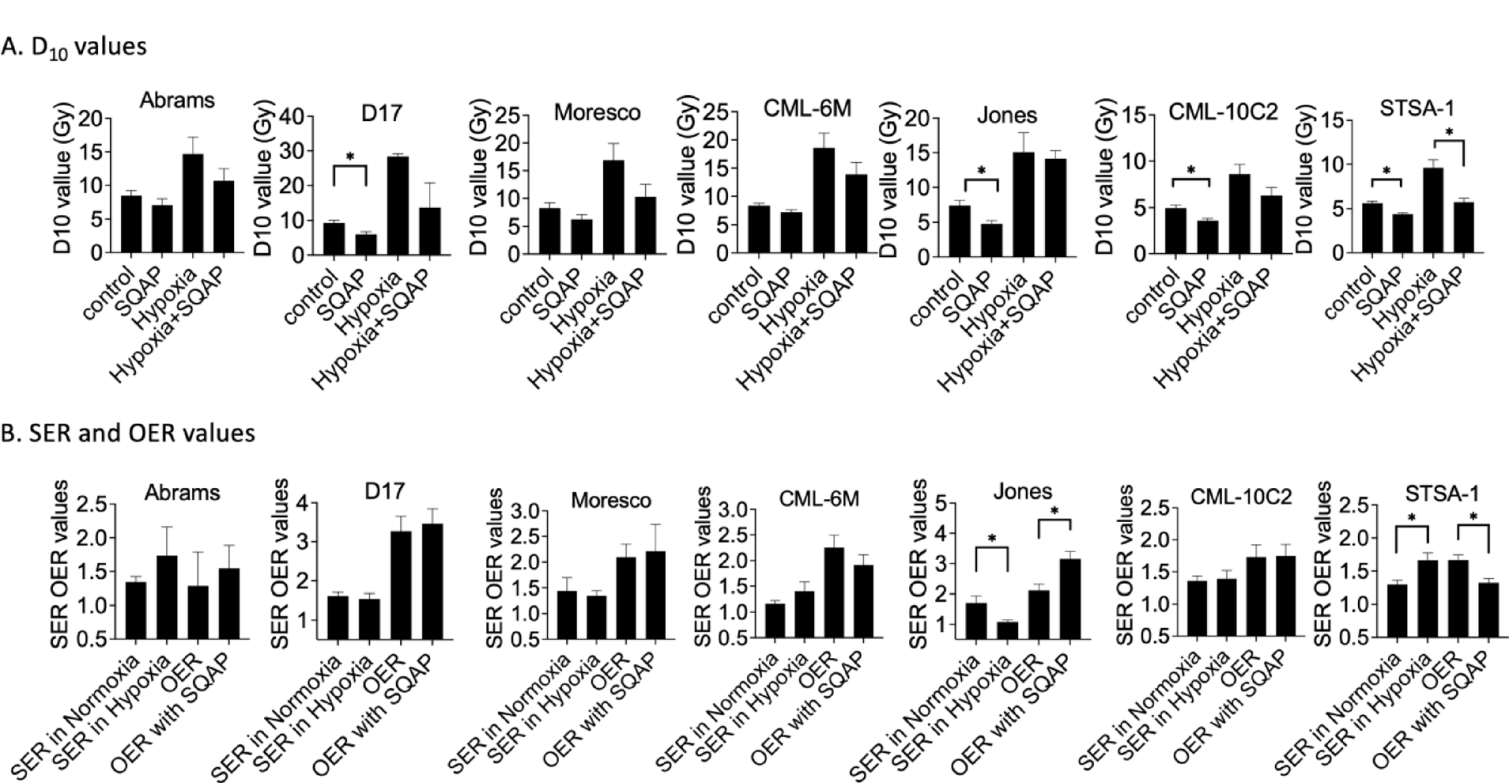
Summary of D_10_ values, SER and OER of seven canine cancer cell lines irradiated with gamma-rays. D_10_ value, a dose to achieve 10% cell survival; OER, oxygen enhancement ratio; SER, sensitization enhancement ratio. Error bars indicate standard error of the mean. * symbols indicate statistically significant differences (*P* < 0.05).

The cytotoxicity of continuous exposure of SQAP in the seven canine cancer cell lines were tested first (Fig. 1A). Each cell line has different cytotoxicity against SQAP treatment, and all cell lines showed tolerance at 30 µM of SQAP but minimal cell viability at 60 µM. Therefore, 30 µM of SQAP were selected for the following radiation experiments with canine cancer cells. Cell survival curves were generated from colony formation assays of each cell line with SQAP in combination with gamma-ray exposure (Fig. 1B,C). SQAP treatment induced radiosensitization to gamma-ray exposure under both normoxic and hypoxia conditions in three canine osteosarcoma cell lines (Abrams, D17 and Moresco), three canine melanoma cell lines (CML-6M, Jones and CML-10C2) and one canine soft-tissue sarcoma cell lines (STSA-1) (Fig. 1B,C). The results of the clonogenic survival assays are summarized in Fig. 2A,B. The D_10_ values (doses to achieve 10% cell survival) for the seven cell lines ranged from 4.95 to 9.28 Gy in normoxia, 3.60 to 7.24 Gy in normoxia with SQAP treatment, 8.58 to 28.4 Gy in hypoxia, and 5.73 to 14.1 Gy in hypoxia with SQAP treatment. SQAP treatment reduced D_10_ values in all tested cell lines. Especially, D17, Jones, CML-10C2, and STSA-1 in normoxia and STSA-1 in hypoxia presented significant reduction of D_10_ values and radiosensitization by SQAP treatment (*p* < 0.05) (Fig. 2A). The calculated sensitizer enhancement ratios (SER) ratios and oxygen enhancement ratios (OER) ratios at D_10_ are shown in Fig. 2B. For each canine cancer cell line, the SER values in normoxia and in hypoxia ranged approximately from 1.2 to 1.7 and from 1.1 to 1.7, respectively, and varied among cell lines. Changes in SER values did not show clear trend. Significant differences were observed in Jones and STSA-1 between normoxia and hypoxia conditions. As expected, the cells in hypoxia were more resistant to ionizing radiation than in normoxia. The radioresistance due to hypoxic condition of each cell line was quantified as OER. Changes in OER values with and without SQAP treatment were not observed. In general, SQAP treatment increased cellular radiosensitivity under both normoxic and hypoxia conditions, and did not affect selectively hypoxic cells.

### SQAP sensitizes canine cancer cells to clinical chemotherapeutic agents in vitro

**Fig. 3:**
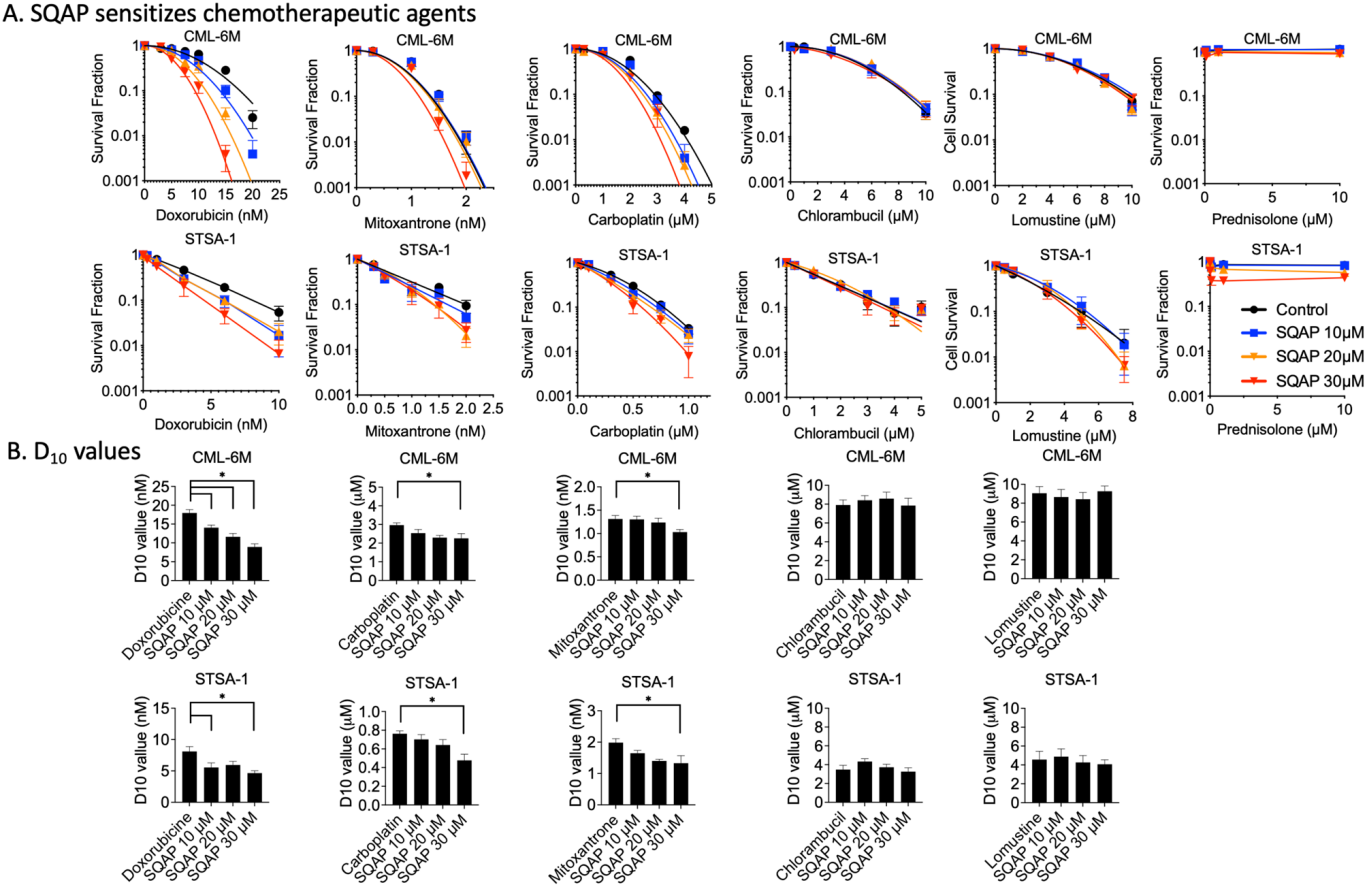
SQAP sensitizes canine cancer cells to chemotherapeutic agents. (**A**) Clonogenic survival of CML-6M and STSA-1 cells treated with 10, 20, 30 µM SQAP and chemotherapeutic agents. (**B**) D_10_ values for chemotherapeutic agents co-treated with SQAP in two canine cancer cell lines. D_10_ values for Prednisolone were not available due to low cytotoxicity. At least three independent experiments were carried out. Error bars indicate standard error of the mean. * symbols indicate statistically significant differences (*P* < 0.05).

If DNA repair inhibition plays a significant role in the radiosensitizing effect of SQAP, SQAP may also affect the cytotoxicity of chemotherapeutic agents known to induce DNA damage. We examined the effect of SQAP on cell survival in two cell lines, CML-6M and STSA-1, using colony formation assays (Fig. 3A). Six common chemotherapy agents applied in the treatment in veterinary cancer medicine were used, including doxorubicin (DOX), carboplatin (CARBO), mitoxantrone (MTX), chlorambucil (CBL), lomustine (CCNU) and prednisolone. Among them doxorubicin showed the most dramatic sensitization by 30 µM of SQAP, with the D_10_ decreasing from 18.0 to 8.94 nM in CML-6M cells and 8.11 to 4.66 nM in STSA-1 cells (Fig. 3B). Doxorubicin is a topoisomerase II inhibitor, known to induce DNA double-strand breaks^31^. Mitoxantrone, another topoisomerase II inhibitor, and carboplatin, a platinum-containing DNA-crosslinking agent, showed significant sensitization at 30 µM of SQAP treatment, which is same concentrations as radiosensitization test. Furthermore, the alkylating agents chlorambucil (CBL) and lomustine (CCNU), as well as the anti-inflammatory glucocorticoid prednisolone, showed no sensitization when combined with SQAP treatment in CML-6M and STSA-1 cells.

### Timing dependent radiosensitization and broad chemosensitization by SQAP

**Fig. 4:**
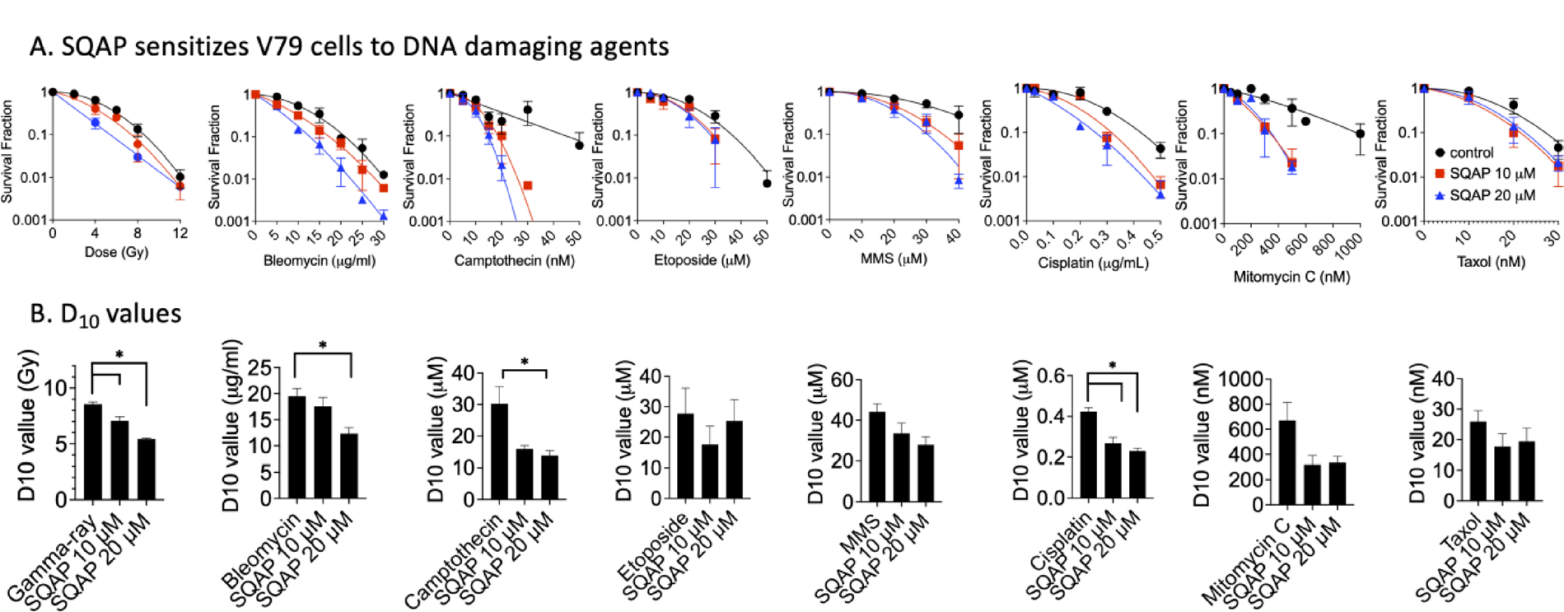
SQAP sensitize Chinese hamster V79 cells to ionizing radiation and DNA damaging drugs. (**A**) Cell survival curves of V79 wild type treated to various DNA damaging agents in combination with SQAP. (**B**) D_10_ values for radiation and chemotherapeutic agents co-treated with SQAP in V79 cells. At least three independent experiments were carried out. Error bars indicate standard error of the mean. * symbols indicate statistically significant differences (*P* < 0.05).

To further confirm the radio- and chemosensitization effects of SQAP observed in canine cancer cells, we tested Chinese hamster V79 cells with cytotoxic agents of well-known mechanisms, including gamma-rays, bleomycin, camptothecin, etoposide, methyl methanesulfornate (MMS), mitomycin C (MMC), cisplatin, and Taxol (Fig. 4A). Based on the cytotoxicity profile of V79 cells, SQAP concentrations of 10 and 20 µM were selected, which maintained sufficient cell viability while allowing us to evaluate concentration-dependent effects. Under normoxic condition, SQAP enhanced radiosensitivity in V79 cells, with a sensitization enhancement ratio (SER) of 1.3. A broad range of chemosensitization effects was observed in V79 cells. SQAP increased the cytotoxicity of not only camptothecin (topoisomerase I inhibitor) and etoposide (topoisomerase II inhibitor), but also MMS (alkylating agent), MMC (DNA-crosslinking agent), cisplatin (platinum-containing DNA-crosslinking agent), bleomycin (DNA DSB inducer) and Taxol (microtubule targeting agent). Additionally, D_10_ values, radiation doses and drug concentrations to reduce cell survival to 10%, were analyzed (Fig. 4B). Gamma-rays, bleomycin, Camptothecin, and Cisplatin showed significant reduction of D_10_ values with SQAP treatment. This DNA damage specific sensitization was matched with the sensitization observed in canine cancer cells (Fig. 3).

**Fig. 5:**
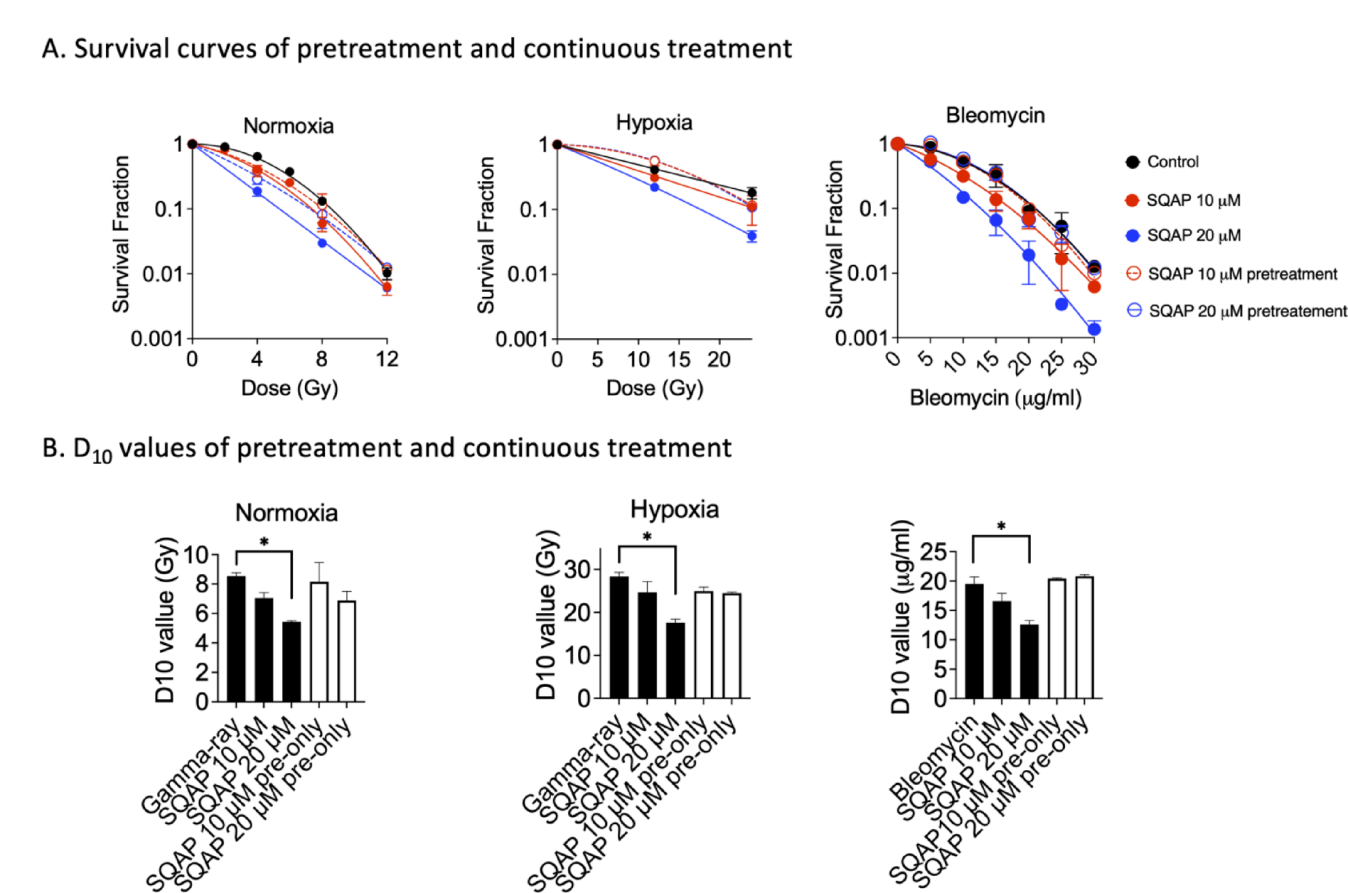
SQAP sensitizes Chinese hamster V79 cells to ionizing radiation and bleomycin under continuously treatment but not under pretreatment. (**A**) Cell survival curves of V79 cells exposed to ionizing radiation under normoxic and hypoxic conditions or treated with bleomycin. SQAP was administered either as continuous treatment or as pretreatment prior to DNA-damaging agent exposure. (**B**) D_10_ values for radiation and bleomycin under different SQAP treatment conditions. Data represent at least three independent experiments. Error bars indicate standard error of the mean. * symbols indicate statistically significant differences (*P* < 0.05).

To clarify the discrepancy with previous reports showing no radiosensitization by SQAP in vitro, we tested the effect of treatment timing in V79 cells under both normoxic and hypoxic conditions (Fig. 5A). Cells were treated with SQAP either only prior to irradiation or continuously before and after irradiation. Radiosensitization was observed only with continuous SQAP treatment, whereas pretreatment alone did not enhance radiation sensitivity as observed in significant reduction of D_10_ values (Fig. 5B). This timing-dependent effect was further confirmed with bleomycin. SQAP sensitized cells to bleomycin only when administrated concomitantly, but not when cells were pretreated without simultaneous drug exposure (Fig. 5A,B).

### SQAP disrupts DNA repair capacity

**Fig. 6:**
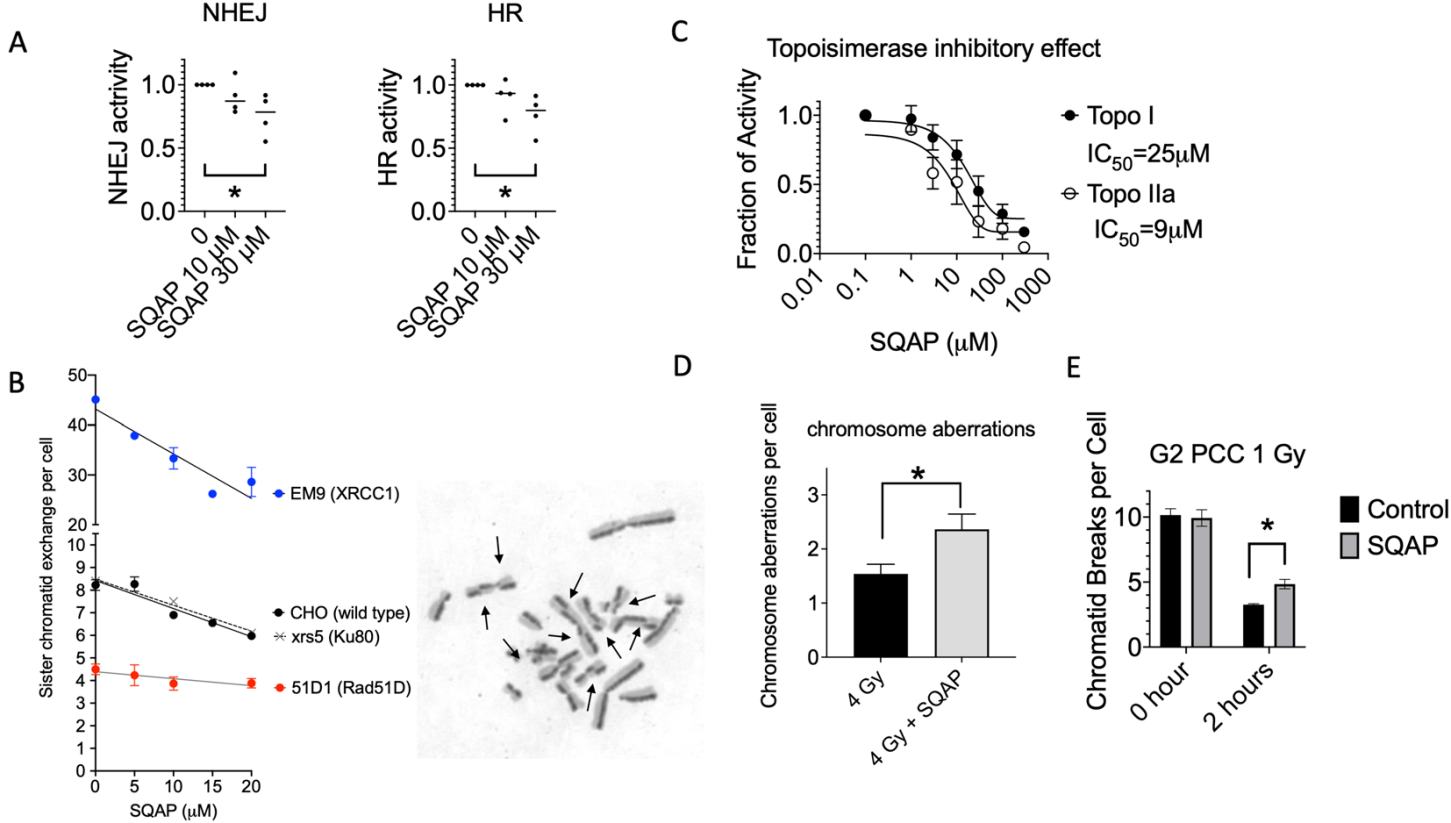
SQAP inhibits DNA repair and affects chromosomal integrity. (**A**) NHEJ and HR activities measured with HeLa DR-GFP and HeLa EJ5-GFP reporter systems. (**B**) Sister chromatid exchange formation (SCE) formation in CHO (wild-type), xrs-5 (Ku80-deficient), 51D1 (Rad51D-deficient) and EM9 (XRCC1-deficient) cells. Representative SCEs in CHO cells are indicated by arrows. (**C**) Topoisomerase activity inhibition assay in the presence of SQAP. Cell-free biochemical assays were performed at the broader concentrations without the confounding effects of cytotoxicity. (**D**) Analysis of post-mitotic chromosome aberrations following of G1-phase irradiated CHO cells. (**E**) Premature chromosome condensation assay for the analysis of radiation-induced chromatin breaks in G2-phase CHO cells. At least three independent experiments were carried out. Error bars indicate standard error of the mean. *, *p* < 0.05 over control.

To further identify potential mechanisms of radiosensitization we utilized several assays to determine DNA repair inhibition by SQAP. SQAP treatment reduced GFP positive cell fractions in HeLa EJ5-GFP and DR-GFP cells and it indicated that SQAP inhibited NHEJ and HR activity in HeLa cells in a dose dependent manner (Fig. 6A). Both NHEJ and HR activities were significantly reduced with 30 µM of SQAP treatment (*p* < 0.05). Sister chromatid exchange represents chromosomal recombination frequency. SQAP treatment reduced the spontaneous sister chromatid exchange formation in CHO wild type, EM9 (XRCC1 mutant), xrs5 (Ku80 mutant) with SQAP dose dependent manner between 5 and 20 µM. Statistical significant reduction was observed from 10 µM and above. On the other hand, 51D1 (rad51d mutant, homologous recombination (HR) repair deficient) presented spontaneously fewer SCE frequency and showed no reduction in the tested non toxic SQAP concentrations (Fig. 6B). In vitro topoisomerase assay measures in vitro decatenation of plasmids by topoisomerases in presence of a broad range of SQAP concentrations. It revealed that SQAP has a function of novel topoisomerase inhibitor. SQAP inhibited both Topo I and Topo II alpha activities to decatenate plasmids with increasing concentrations. The degree of inhibition was greater in Topo II alpha, with an IC_50_ of 9 µM compared with 25 µM for Topo I (Fig. 6C).

### Cytogenetic analysis of SQAP induced radiosensitization in Chinese hamster cells

SQAP treatment enhanced radiation-induced chromosome aberration formation in CHO cells (Fig. 6D). Irradiation of 4 Gy induced approximately 1.5 chromosome-type aberrations in G1-irradiated cells. SQAP treatment enhanced this to approximately 2.4 with statistical significance (*p* < 0.05). Chromosome aberrations were also analyzed in a different method, G2-premature chromosome condensation (PCC) assay (Fig. 6E). The number of G2-PCC chromatid breaks immediately after 1 Gy irradiation in SQAP-treated CHO cells was approximately 10, the same as that in CHO cells not treated with SQAP. Therefore, SQAP treatment did not increase initial radiation-induced chromosome aberrations. After 2 h, SQAP treatment statistically significantly increased the residual PCC damage with residual PCC levels of approximately 3.3 without SQAP and 4.8 with SQAP.

## Discussion

Previous studies, mainly using xenograft models, have shown that SQAP exerts radiosensitizing effects by modulating the hypoxic tumor microenvironment. Our results extend these findings by demonstrating that SQAP enhances the cytotoxic effects of both radiation and DNA-damaging agents across multiple cell types, including canine cancer cells and Chinese hamster cells. In canine cancer cells, radiosensitization was observed under both normoxic and hypoxic conditions, and sensitization to chemotherapeutic agents such as doxorubicin and mitoxantrone was also evident (Figs. 1, 2 and 3). Similarly, Chinese hamster cells treated concomitantly with SQAP exhibited enhanced sensitivity to a broad range of DNA-damaging agents, including ionizing radiation, bleomycin, camptothecin, etoposide, and platinum compounds (Fig. 4).

These results indicate that SQAP does not act as a hypoxia-selective cytotoxins or oxygen mimics^32^. Rather, its radiosensitizing and chemosensitizing effects are likely mediated through modulation of DNA repair pathways. Continuous exposure to SQAP before and after irradiation, as used in our study^12,33^, induced radiosensitization under both normoxic and hypoxic conditions, whereas pretreatment alone was insufficient (Fig. 5). This continuous exposure better reflects in vivo pharmacokinetics, as SQAP is rapidly cleared from normal tissues but persists in tumors for several hours^34^.

Double strand breaks (DSBs) are the most cytotoxic form of DNA damage induced by ionizing radiation and many chemotherapeutic agents^15^. Using human cell-based reporter assays, we found that SQAP inhibits both canonical DSB repair pathways, NHEJ and HR (Fig. 6A). Additionally, sister chromatid exchange (SCE) assays demonstrated that HR-proficient CHO cells exhibited reduced SCE frequency upon SQAP treatment, whereas HR deficient cells were unaffected (Fig. 6B), indicating impairment of HR^35^. Consistent with the dependence of SCE on HR activity, spontaneous SCE frequency is normally high in EM9 (XRCC1, base excision repair deficient mutant) and BLM-deficient cells, and low in HR-deficient cells^19,36^. Accordingly, CHO wild type, xrs5 (Ku80, NHEJ repair deficient mutant, and EM9 cells all showed reduced SCE frequency upon SQAP treatment, while HR-deficient Rad51D mutant cells did not respond. Moreover, SQAP enhanced chromosome damage resulting from unrepaired or misrepaired DSBs (Fig. 6D and E), leading to increased cytotoxicity across diverse DNA-damaging agents^37^. By inhibiting both NHEJ and HR, SQAP effectively amplifies cellular radiosensitivity and chemosensitivity.

**Fig. 7:**
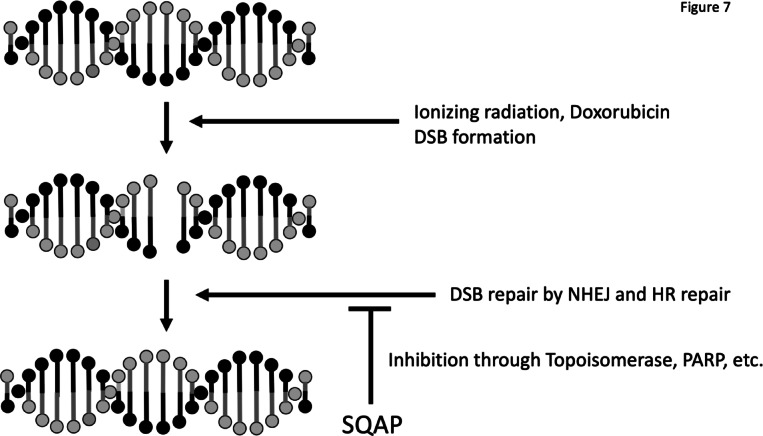
A schematic summary of the proposed mechanisms by which SQAP mediates radiosensitization and chemosensitization, integrating the key findings from our study.

The precise mechanisms underlying SQAP mediated DNA repair inhibition remain to be fully defined. Multiple proteins and factors influence NHEJ and HR, including DNA damage signaling, chromatin structure, and cell cycle stage^38,39^. Notably, we previously identified that SQAP inhibits PARP and PARG, inducing synthetic lethality in HR deficient cells^13^. PARP and PARG regulate single strand break repairs, and contributes to both NHEJ and HR^40^, and their inhibition sensitizes cells to DNA damaging agents^41^. These findings are consistent with the reporter assays and SCE results presented here (Fig. 6). Additional in vitro assays demonstrated that SQAP disrupts Topoisomerase I and II alpha activities, with Topoisomerase II alpha showing the lowest IC50 (9 µM), suggesting it as a potential key target^42,43^. HDAC1, another previously reported SQAP target^11^, has been implicated in radiosensitization under hypoxia, however, we didn’t observe hypoxia selective radiosensitization in vitro (Fig. 1A). Collectively, our results point to NHEJ, HR, and Topoisomerase II alpha as primary contributors to SQAP-mediated radionsensitization and chemosensitization (Fig. 7), though further investigation is warranted.

In clinical aspect, SQAP enhanced the cytotoxicity of doxorubicin, carboplatin, and mitoxantrone in canine cancer cells (Fig. 3). Doxorubicin functions as a topoisomerase II inhibitor and intercalates DNA, generating strand breaks and oxidative stress^31,44^. SQAP may allow reduced dosing of doxorubicin while maintaining efficacy, potentially mitigating dose-limiting cardiotoxicity^45^. Cisplatin and carboplatin also showed sensitization in both canine and Chinese hamster cells. In contrast, other chemotherapeutic agents, such as chlorambucil and lomustine, did not show enhanced cytotoxicity in the canine cell lines, possibly reflecting tumor specific differences in DNA repair capacity^46^. These results highlight the need for further evaluation across additional cancer lines and suggest doxorubicin as a promising candidate for combination therapy with SQAP.

Taken together, our study demonstrated that SQAP enhances radiosensitivity and chemosensitivity by impairing DNA repair pathways, including NHEJ and HR, across species and cell types. Continuous exposure in vitro more accurately reflects in vivo pharmacokinetics, supporting translational relevance. In addition to the previously reported effects on the hypoxic tumor microenvironment, our findings identify potential molecular targets, Topoisomerase II alpha for further mechanistic studies. Collectively, these results position SQAP as a versatile radiosensitizer and chemosensitizer with potential clinical applicability for improving the efficacy of radiotherapy and chemotherapy.

## Data Availability

All data generated or analyzed during this study are included in this published article.

## References

[1] Hanashima, S. et al. Synthesis of sulfoquinovosylacylglycerols, inhibitors of eukaryotic DNA polymerase alpha and beta. *Bioorg. Med. Chem.* **9**, 367–376. 10.1016/s0968-0896(00)00252-2 (2001). 11249129 10.1016/s0968-0896(00)00252-2

[2] Sahara, H. et al. Anti-tumor effect of chemically synthesized sulfolipids based on sea urchin’s natural sulfonoquinovosylmonoacylglycerols. *Jpn. J. Cancer Res. Gann*. **93**, 85–92. 10.1111/j.1349-7006.2002.tb01204.x (2002). 11802812 10.1111/j.1349-7006.2002.tb01204.x PMC5926865

[3] Sakimoto, I. et al. Alpha-sulfoquinovosylmonoacylglycerol is a novel potent radiosensitizer targeting tumor angiogenesis. *Cancer Res.* **66**, 2287–2295. 10.1158/0008-5472.CAN-05-2209 (2006). 16489033 10.1158/0008-5472.CAN-05-2209

[4] Ohta, K. et al. Remodeling of the tumor microenvironment by combined treatment with a novel radiosensitizer, α-sulfoquinovosylmonoacylglycerol (α-SQMG) and X-irradiation. *Anticancer Res.* **30**, 4397–4404 (2010). 21115885

[5] Takakusagi, Y. et al. A multimodal molecular imaging study evaluates pharmacological alteration of the tumor microenvironment to improve radiation response. *Cancer Res.* **78**, 6828–6837. 10.1158/0008-5472.CAN-18-1654 (2018). 30301838 10.1158/0008-5472.CAN-18-1654 PMC8127870

[6] Inamasu, E. et al. Anticancer agent alpha-sulfoquinovosyl-acylpropanediol enhances the radiosensitivity of human malignant mesothelioma in nude mouse models. *J. Radiat. Res.* **63**, 19–29. 10.1093/jrr/rrab090 (2022). 34738103 10.1093/jrr/rrab090 PMC8776698

[7] Sawada, Y. et al. Sulfoquinovosylacylpropanediol is a novel potent radiosensitizer in prostate cancer. *Int. J. Urol. Off. J. Jpn. Urol. Assoc.* **22**, 590–595. 10.1111/iju.12753 (2015). 10.1111/iju.12753 25781902

[8] Yoshioka, C. et al. Sulfoquinovosyl acyl panediol (SQAP) as a radiation sensitizer for dogs with tumors: A pilot study. *J. Azabu Univ.* **31**, 53–59(2020).

[9] Kutara, K. et al. The outcome and CT findings of low-dose intensity modulated radiation therapy with SQAP in a cat with thymoma. *Vet. Sci.* **7**. 10.3390/vetsci7040203 (2020). 10.3390/vetsci7040203 PMC7765136 33327647

[10] Beckers, C., Pruschy, M. & Vetrugno, I. Tumor hypoxia and radiotherapy: A major driver of resistance even for novel radiotherapy modalities. *Semin Cancer Biol.* **98**, 19–30. 10.1016/j.semcancer.2023.11.006 (2024). 38040401 10.1016/j.semcancer.2023.11.006

[11] Kawakubo, H. et al. SQAP, an acyl sulfoquinovosyl derivative, suppresses expression of histone deacetylase and induces cell death of cancer cells under hypoxic conditions. *Biosci. Biotechnol. Biochem.* **85**, 85–91. 10.1093/bbb/zbaa015 (2021). 33577659 10.1093/bbb/zbaa015

[12] Gerelchuluun, A. et al. Histone deacetylase inhibitor induced radiation sensitization effects on human cancer cells after photon and Hadron radiation exposure. *Int. J. Mol. Sci.* **19**10.3390/ijms19020496 (2018). 10.3390/ijms19020496 PMC5855718 29414878

[13] Maeda, J., Shellenberger, K. D., Kurihara, W., Haga, T. & Kato, T. A. Sulfoquinovosyl acylpropanediol (SQAP): Inhibition of poly(ADP-ribose) metabolism and enhanced cytotoxicity in homologous recombination repair-deficient Chinese hamster-derived cells. *Mutat. Res. Genetic Toxicol. Environ. Mutagen.* **892**, 503703. 10.1016/j.mrgentox.2023.503703 (2023). 10.1016/j.mrgentox.2023.503703 37973295

[14] Izaguirre-Carbonell, J. et al. Novel anticancer agent, SQAP, binds to focal adhesion kinase and modulates its activity. *Sci. Rep.* **5**, 15136. 10.1038/srep15136 (2015). 26456697 10.1038/srep15136 PMC4601023

[15] Iliakis, G. et al. Mechanisms of DNA double strand break repair and chromosome aberration formation. *Cytogenet. Genome Res.* **104**, 14–20 (2004). 15162010 10.1159/000077461

[16] Khanna, K. K. & Jackson, S. P. DNA double-strand breaks: Signaling, repair and the cancer connection. *Nat. Genet.* **27**, 247–254. 10.1038/85798 (2001). 11242102 10.1038/85798

[17] Mladenov, E., Magin, S., Soni, A. & Iliakis, G. DNA double-strand break repair as determinant of cellular radiosensitivity to killing and target in radiation therapy. *Front. Oncol.* **3**, 113. 10.3389/fonc.2013.00113 (2013). 23675572 10.3389/fonc.2013.00113 PMC3650303

[18] Biau, J., Chautard, E., Verrelle, P. & Dutreix, M. Altering DNA repair to improve radiation therapy: Specific and multiple pathway targeting. *Front. Oncol.* **9**, 1009. 10.3389/fonc.2019.01009 (2019). 31649878 10.3389/fonc.2019.01009 PMC6795692

[19] Thompson, L. H. et al. A CHO-cell strain having hypersensitivity to mutagens, a defect in DNA strand-break repair, and anextraordinary baseline frequency of sister-chromatid exchange. *Mutat. Res.* **95**, 427–440 (1982). 6889677 10.1016/0027-5107(82)90276-7

[20] Jeggo, P. A. & Kemp, L. M. X-ray-sensitive mutants of Chinese hamster ovary cell line. Isolation and cross-sensitivity to other DNA-damaging agents. *Mutat. Res.* **112**, 313–327 (1983). 6197643 10.1016/0167-8817(83)90026-3

[21] Hinz, J. M. et al. Repression of mutagenesis by Rad51D-mediated homologous recombination. *Nucleic Acids Res.* **34**, 1358–1368 (2006). 16522646 10.1093/nar/gkl020 PMC1390685

[22] Maeda, J. et al. Intrinsic radiosensitivity and cellular characterization of 27 canine cancer cell lines. *PLoS One*. **11**, e0156689. 10.1371/journal.pone.0156689 (2016). 27257868 10.1371/journal.pone.0156689 PMC4892608

[23] Cartwright, I. M. et al. DNA repair deficient Chinese hamster ovary cells exhibiting differential sensitivity to charged particle radiation under aerobic and hypoxic conditions. *Int. J. Mol. Sci.* **19**. 10.3390/ijms19082228 (2018). 10.3390/ijms19082228 PMC6121575 30061540

[24] Maeda, J. et al. Natural and glucosyl flavonoids inhibit poly(ADP-ribose) polymerase activity and induce synthetic lethality in BRCA mutant cells. *Oncol. Rep.* **31**, 551–556. 10.3892/or.2013.2902 (2014). 24317580 10.3892/or.2013.2902 PMC3896521

[25] Pierce, A. J., Johnson, R. D., Thompson, L. H. & Jasin, M. XRCC3 promotes homology-directed repair of DNA damage in mammalian cells. *Genes Dev.* **13**, 2633–2638 (1999). 10541549 10.1101/gad.13.20.2633 PMC317094

[26] Bennardo, N., Cheng, A., Huang, N. & Stark, J. M. Alternative-NHEJ is a mechanistically distinct pathway of mammalian chromosome break repair. *PLoS Genet.* **4**, e1000110. 10.1371/journal.pgen.1000110 (2008). 18584027 10.1371/journal.pgen.1000110 PMC2430616

[27] Bennardo, N., Gunn, A., Cheng, A., Hasty, P. & Stark, J. M. Limiting the persistence of a chromosome break diminishes its mutagenic potential. *PLoS Genet.* **5**, e1000683. 10.1371/journal.pgen.1000683 (2009). 19834534 10.1371/journal.pgen.1000683 PMC2752804

[28] Terasima, T. & Tolmach, L. J. Changes in x-ray sensitivity of HeLa cells during the division cycle. *Nature* **190**, 1210–1211 (1961). 13775960 10.1038/1901210a0

[29] Kato, T. A. Human lymphocyte metaphase chromosome preparation for radiation-induced chromosome aberration analysis. *Methods Mol. Biol.* **1–6**. 10.1007/978-1-4939-9432-8_1 (1984). (2019). 10.1007/978-1-4939-9432-8_1 31267414

[30] Gotoh, E., Asakawa, Y. & Kosaka, H. Inhibition of protein-serine threonine phosphatases directly induces premature chromosome condensation in mammalian somatic-cells. *Biomed. Res. Tokyo* **16**, 63–68 (1995).

[31] Kciuk, M. et al. Doxorubicin—An agent with multiple mechanisms of anticancer activity. *Cells* **12**. 10.3390/cells12040659 (2023). 10.3390/cells12040659 PMC9954613 36831326

[32] Gong, L., Zhang, Y., Liu, C., Zhang, M. & Han, S. Application of radiosensitizers in cancer radiotherapy. *Int. J. Nanomed.* **16**, 1083–1102. 10.2147/IJN.S290438 (2021). 10.2147/IJN.S290438 PMC7886779 33603370

[33] Okayasu, R., Suetomi, K. & Ullrich, R. L. Wortmannin inhibits repair of DNA double-strand breaks in irradiated normal human cells. *Radiat. Res.* **149**, 440–445 (1998). 9588354

[34] Ruike, T. et al. Distribution and metabolism of (14)C-sulfoquinovosylacylpropanediol ((14)C-SQAP) after a single intravenous administration in tumor-bearing mice. *Xenobiotica* **49**, 346–362. 10.1080/00498254.2018.1448949 (2019). 29543539 10.1080/00498254.2018.1448949

[35] Wilson, D. M., Thompson, L. H. & III & Molecular mechanisms of sister-chromatid exchange. *Mutat. Res.* **616**, 11–23 (2007). 17157333 10.1016/j.mrfmmm.2006.11.017

[36] Chaganti, R. S., Schonberg, S. & German, J. Manifold increase in sister chromatid exchanges in blooms syndrome lymphocytes. *Proc. Natl. Acad. Sci. U.S.A.* **71**, 4508–4512. 10.1073/pnas.71.11.4508 (1974). 4140506 10.1073/pnas.71.11.4508 PMC433916

[37] Reuvers, T. G. A., Kanaar, R. & Nonnekens, J. D. N. A. Damage-inducing anticancer therapies: From global to precision damage. *Cancers* **12** (2020). 10.3390/cancers12082098 10.3390/cancers12082098 PMC7463878 32731592

[38] Shrivastav, M., De Haro, L. P. & Nickoloff, J. A. Regulation of DNA double-strand break repair pathway choice. *Cell. Res.* **18**, 134–147. 10.1038/cr.2007.111 (2008). 18157161 10.1038/cr.2007.111

[39] Kanev, P. B., Atemin, A., Stoynov, S. & Aleksandrov, R. PARP1 roles in DNA repair and DNA replication: The basi(c)s of PARP inhibitor efficacy and resistance. *Semin Oncol.* **51**, 2–18. 10.1053/j.seminoncol.2023.08.001 (2024). 37714792 10.1053/j.seminoncol.2023.08.001

[40] Ray Chaudhuri, A. & Nussenzweig, A. The multifaceted roles of PARP1 in DNA repair and chromatin remodelling. *Nat. Rev. Mol. Cell. Biol.* **18**, 610–621. 10.1038/nrm.2017.53 (2017). 28676700 10.1038/nrm.2017.53 PMC6591728

[41] Harrision, D., Gravells, P., Thompson, R. & Bryant, H. E. Poly(ADP-Ribose) glycohydrolase (PARG) versus Poly(ADP-Ribose) polymerase (PARP)—Function in genome maintenance and relevance of inhibitors for anti-cancer therapy. *Front. Mol. Biosci.* **7**, 191. 10.3389/fmolb.2020.00191 (2020). 33005627 10.3389/fmolb.2020.00191 PMC7485115

[42] Chen, A. Y., Choy, H. & Rothenberg, M. L. DNA topoisomerase I-targeting drugs as radiation sensitizers. *Oncology-Ny* **13**, 39–46 (1999). 10550825

[43] Kim, J. S., Amorino, G. P., Pyo, H., Cao, Q. W. & Choy, H. Radiation enhancement by the combined use of topoisomerase I inhibitors, RFS-2000 or CPT-11, and topoisomerase II inhibitor Etoposide in human lung cancer cells. *Radiother. Oncol.* **62**, 61–67. 10.1016/S0167-8140(01)00465-0 (2002). doi:Pii S0167-8140(01)00465-0. 11830313 10.1016/s0167-8140(01)00465-0

[44] Nitiss, J. L. Targeting DNA topoisomerase II in cancer chemotherapy. *Nat. Rev. Cancer* **9**, 338–350. 10.1038/nrc2607 (2009). 19377506 10.1038/nrc2607 PMC2748742

[45] Rawat, P. S., Jaiswal, A., Khurana, A., Bhatti, J. S. & Navik, U. Doxorubicin-induced cardiotoxicity: An update on the molecular mechanism and novel therapeutic strategies for effective management. *Biomed. Pharmacother*. **139**, 111708. 10.1016/j.biopha.2021.111708 (2021). 34243633 10.1016/j.biopha.2021.111708

[46] Hopkins, J. L., Lan, L. & Zou, L. DNA repair defects in cancer and therapeutic opportunities. *Genes Dev.* **36**, 278–293. 10.1101/gad.349431.122 (2022). 35318271 10.1101/gad.349431.122 PMC8973847

